# Zero-tillage is a proven technology for sustainable wheat intensification in the Eastern Indo-Gangetic Plains: what determines farmer awareness and adoption?

**DOI:** 10.1007/s12571-017-0707-x

**Published:** 2017-07-22

**Authors:** Alwin Keil, Alwin D souza, Andrew McDonald

**Affiliations:** 1 CIMMYT-India, CG Block, National Agricultural Science Centre (NASC) Complex, DPS Marg, New Delhi 110012, India; 2 CIMMYT-Nepal, Nepal Agricultural Research Council (NARC), Agricultural Botany Division, Khumultar, Kathmandu, Nepal

**Keywords:** Zero-tillage, Technology adoption, Non-exposure bias, Social network effects, Bihar

## Abstract

In India, there is increasing recognition among policy-makers of the largely untapped potential of the Eastern Indo-Gangetic Plains (IGP) for meeting state- and national-level food needs. Zero-tillage (ZT) is a proven technology for enhancing wheat productivity and, hence, food security in the IGP, while reducing production costs – a ‘win-win’ scenario that should support rapid technology scaling even though adoption remains modest to date. In order to inform policies and derive recommendations for a more effective extension strategy, this study investigated determinants of ZT adoption in the Eastern IGP using a random sample of 1000 wheat-growing households from Bihar, stratified by ZT adoption status. We corrected for potential non-exposure bias by using a two-stage estimation procedure that differentiates between factors affecting farmers’ awareness of ZT and those affecting adoption conditional on awareness. Owing to the relatively nascent stage of ZT diffusion in the area, we emphasized the role of information in the adoption process, including social networks, whereby we allowed for endogenous and exogenous network effects. Only 44% of sample households knew about ZT technology, and there was substantial scale bias in favor of larger scale farmers both with respect to awareness and adoption. Both the adoption behavior and characteristics of the respondents’ network members influenced their own awareness and adoption of ZT, particularly among farmers in the smallest landholding tercile. Farmers valued the time-saving potential of ZT, especially under conditions of increasingly unreliable monsoon rains resulting in a delayed rice crop and, consequently, late establishment of wheat which reduces yield. The fact that most farmers accessed ZT via custom-hire services was accounted for in the model, and the importance of proximate service providers confirmed. We conclude that there is need for further awareness-raising campaigns for ZT technology, where-by, apart from mass media, agricultural extension should use contact farmers belonging to different social strata for effective within-village diffusion of messages, especially to the poorer farmers. The social inclusiveness of ZT use can be enhanced by supporting the emergence of more ZT service providers and by developing business models that lower the transaction costs of servicing smaller farms.

**JEL Classifications** O33 . Q55

## Introduction

The Indo-Gangetic Plains (IGP) are home to more than 20% of the global population, and sustainably enhancing the productivity of the prevailing rice-wheat cropping systems will be of utmost importance for ensuring future food security (Chauhan et al. 2012). The potential to increase yields is particularly large in the Eastern IGP, such as the state of Bihar. On the one hand, Bihar has the lowest cereal yields in the IGP: over the period 2012/13–2013/14, wheat yields averaged 2.34 t ha^−1^, as opposed to 4.79 t ha^−1^ in the Northwestern state of Punjab (MoA 2015). On the other hand, the Eastern IGP has a wealth of under-developed water resources (Aggarwal et al. 2004; DoA^2008^),whereas excessive irrigation has led to dramatic declines in groundwater tables in the Northwest IGP (Humphreys et al. 2010). Consequently, Indian policy makers have turned their attention to meeting both state-level and national food needs through agricultural intensification programs in the East, such as ‘Bringing the Green Revolution to Eastern India’ (BGREI; http://bgrei-rkvy.nic.in). Identifying technical entry points and strengthening support systems for innovations that will contribute to agricultural intensification in a manner that is environmentally sustainable, socioeconomically tenable, and, just as importantly, broadly scalable among smallholders presents a formidable challenge.

Across the IGP, the combination of zero tillage (ZT) and residue retention has been found to have considerable agronomic and economic benefits, while improving the environmental footprint of agriculture by reducing energy costs and raising soil and water quality (Mehla et al. 2000; Erenstein and Laxmi 2008; Chauhan et al. 2012; Gathala et al. 2013). In ZT wheat, agronomic factors leading to productivity advantages are related to (i) time-savings in crop establishment by omitting tillage operations with multiple passes of the tractor to accomplish plowing, harrowing and planking operations; this allows earlier sowing and, hence, reduces risks of terminal heat stress during the grain-filling phase of wheat; (ii) better control of damaging weeds; (iii) better nutrient management; and (iv) water savings (Mehla et al. 2000; Erenstein and Laxmi 2008; Gathala et al. 2013). Reviewing the impacts of ‘conservation tillage’, mostly ZT, on wheat productivity across South Asia, Krishna et al. (2017) found that 13 out of 25 published studies reported a significant yield gain, while none indicated a statistically significant yield loss. Together with documented savings in fuel and crop establishment costs, which have served as the main selling point of ZT technology in the region (Coventry et al. 2015) and outweigh potentially increased expenses for chemical weed control (Keil et al. 2015), there is thus ample evidence that the practice is economically superior to conventional-tillage wheat. While most of the studies were conducted in the Northwestern IGP, there is also empirical evidence of ZT wheat to be an attractive technology for farmers in the poverty-stricken Eastern IGP, where full crop residue retention is not a viable option for most farmers due to the use of residues as fodder. Keil et al. (2015) found that the cost savings and yield gain associated with the use of ZT wheat in Bihar led to a combined economic benefit of approximately 110 US$ ha^−1^ compared to conventionaltillage wheat; for the average ZT wheat adopter in Bihar, the benefits derived from the practice were equivalent to 6% of their total annual household income. ZT-induced wheat yield gains were estimated at 17.4% in the prevailing rice–wheat system with intensive soil puddling in the rice component and only partial retention of rice residues^1^ (Keil et al. 2015).

Despite rich evidence of the economic benefits achieved with ZT wheat, the adoption of the technology has remained slow, with the highest rate estimated at 25%^2^ in the Northwestern IGP, and the lowest, 2%, in the East (Singh et al. 2012). The observed regional differences in the uptake of ZT seemed to be closely associated with the time of introduction of the technology (Singh et al. 2012). In view of the increasing recognition of the largely untapped potential of ZT for meeting state- and national-level food needs, investigating the determinants of farmers’ adoption of ZT in the Eastern IGP is of particular importance. Hereby, two complicating factors have to be taken into consideration: (i) due to significantly higher poverty rates in the East, tractor and ZT drill ownership is not a tenable goal for the large majority of farmers; hence, they rely on custom-hire services to access the technology (Erenstein and Laxmi 2008; Keil et al. 2016); and (ii), compared with the Northwestern IGP, farmers’ awareness of the technology is still low (Singh et al. 2012).

Regarding factor (ii), classical approaches to modeling technology adoption behavior have largely ignored the role of information (Kabunga et al. 2012), concentrating on farmers’ personal characteristics as well as infrastructural conditions as explanatory factors (cf. Feder et al. 1985). However, the role of information is likely to be critical, especially in situations where a technology is relatively new to an area, as is the case with ZT in Bihar, where many farmers have no knowledge of the technology. If lacking awareness or ‘exposure’ to the technology is not accounted for in a model that identifies adoption determinants, estimates may be affected by non-exposure bias. The bias results from the fact that farmers who do not know about a new technology have no chance to adopt it, although they may have adopted if they had been aware of it (Diagne and Demont 2007). Non-exposure bias can arise due to two different sources: first, farmers may differ in their ambitions to search for information about new technologies and their ability and willingness to process such information; hence, exposure is partly by farmers’ choice, entailing potential self-selection bias. Second, farmers may differ in their access to information about new technologies; in particular, so-called ‘progressive’ farmers and communities may be targeted by agricultural development projects or have a higher level of connectivity to state extension or private sector input suppliers, leading to earlier exposure and adoption of new technologies (Diagne and Demont 2007). Since smallholder farmers often have limited access to formal sources of agriculture-related information, such as agricultural extension, adoption studies have become more complex by taking dynamic processes of learning by doing and learning from others into consideration (Foster and Rosenzweig 1995). Relatively recent empirical studies that explored such social learning processes found that for gathering information and making adoption decisions, farmers mainly rely on small individual social networks (Conley and Udry 2001; Bandiera and Rasul 2006; Matuschke and Qaim 2009). As such, farmers may not only be influenced by what their social networks do (endogenous network effect), but also by who they are (exogenous network effect; Manski 2000).

Taking the above considerations into account, the objective of this study was to quantify determinants of ZT adoption using household survey data from Bihar. The study contributes to the existing body of literature in several ways. First, since the use of ZT is a relatively new practice in the Eastern IGP, this is the first such assessment in this environment which differs significantly from the agriculturally more productive states in the Northwestern IGP. Second, owing to the relatively nascent stage of ZT adoption in the area, we emphasize the role of information in the adoption process. We account for the fact that a substantial share of farm households in Bihar are likely unaware of ZT technology by using a two-stage estimation procedure correcting for non-exposure bias. Third, acknowledging the importance of access to information, we explore the role of social networks in technology adoption, allowing for both endogenous and exogenous network effects. Fourth, a unique dataset allows us to account for the presence of a proximate ZT service provider as a crucial prerequisite to the use of the technology.

### Research area, sampling procedure, and data collection

**Fig. 1:**
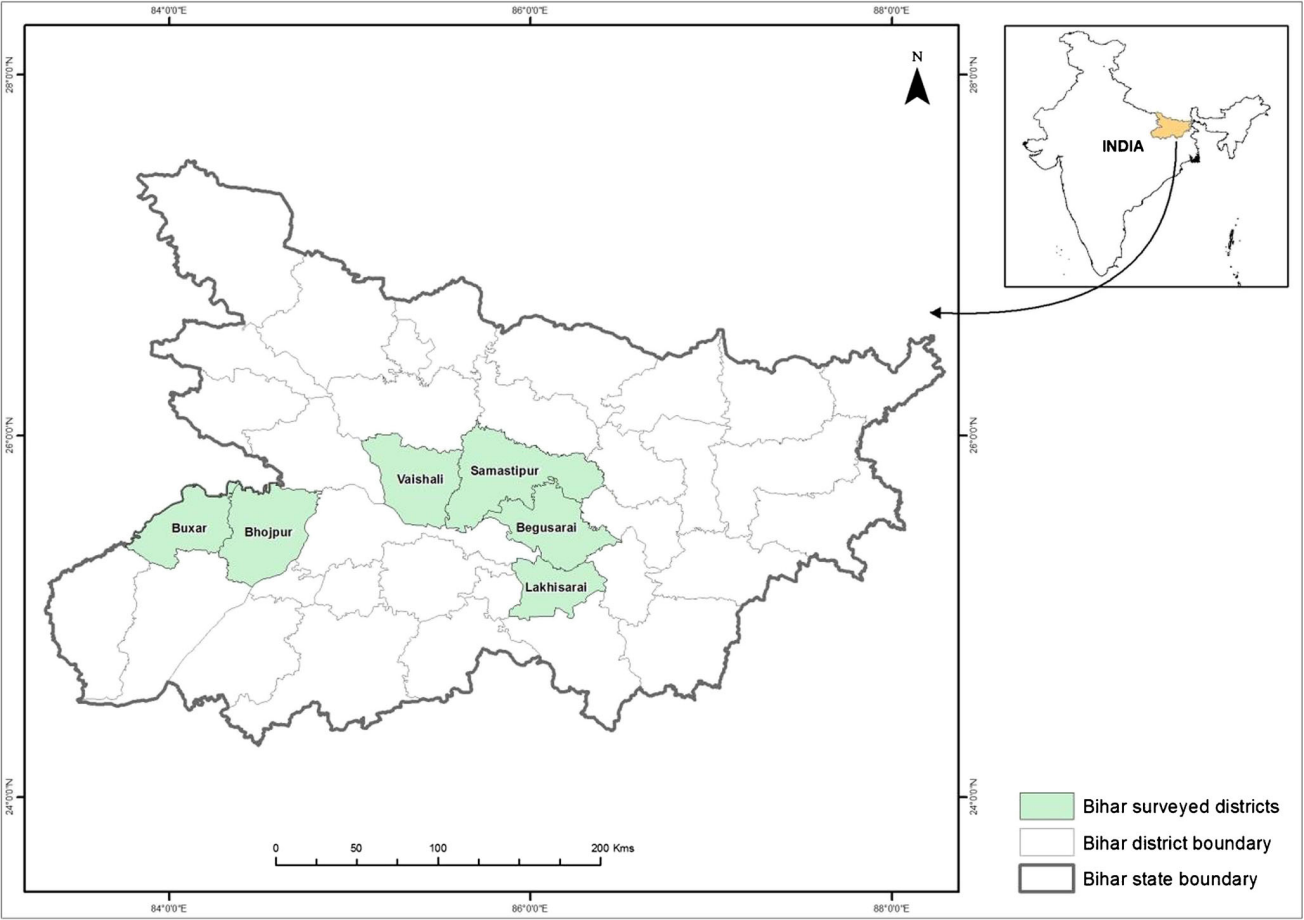
Map of the state of Bihar in northern India, highlighting the survey districts

Agriculture is the main occupation in Bihar with almost 81% of its population engaged, whereas its contribution to State domestic product is merely 42% (DoA 2008). Paddy rice, wheat, pulses, maize, potato, sugarcane, oil seeds, tobacco and jute are the principal crops grown. Although Bihar is endowed with good soil, sufficient rainfall and abundant groundwater, its agricultural productivity is one of the lowest among Indian states (DoA 2008). The research area is composed of six districts in Bihar where the Cereal Systems Initiative for South Asia (CSISA; www.csisa.org) has focused research and outscaling activities for sustainable intensification technologies since 2009 (Fig. 1). Using a cluster sampling approach, data were collected in a random sample of 1000 wheat growing farm households from August to October 2013, whereby the sample was stratified by ZT adoption status. In a first step, 40 villages were randomly selected out of 87 villages with at least 10 ZT users in the target districts, as documented by CSISA. Hereby, the number of research villages per district was proportionate to the distribution of eligible villages, resulting in three research villages each in Begusarai, Lakhisarai and Vaishali districts, six villages each in Buxar and Samastipur, and 19 villages in Bhojpur district. Since reliable household lists were not available, a brief census survey was conducted in each selected village to elicit households’ main occupation, incidence of wheat cultivation (since, in Bihar, ZT is thus far almost exclusively used in wheat), and ZT adoption status to permit sample stratification. As a last step, 10 ZT users and 15 non-users were randomly selected among all wheat growing farm households in each village. This stratified sampling approach is justified since the main objective of the work reported in this paper is not to assess average ZT wheat adoption rates, but factors influencing households’ awareness and adoption of ZT wheat. Given the relatively nascent stage of ZT diffusion in the area, we had to ensure an adequate size of the adopter sub-sample through stratification, which is a common and recommended procedure (cf. Deaton 1997: 13). In Bihar, wheat is by far the most widely grown crop during the winter (*rabi*) season (MoA 2015); based on the village census data, 92.8% of households in our research villages were wheat growers. Therefore, confining the sampling frame to wheat-growing households was not expected to introduce any significant bias in our analysis.

Apart from eliciting information on the respondents’ household and farming activities, survey respondents were also asked to provide some basic information on those three farmers whom they interacted with most frequently about agricultural issues in order to be able to capture potential individual social network effects on ZT adoption. Data were collected from household heads by a team of 18 professional enumerators through structured interviews.^3^ Information was elicited about households’ asset endowment and rice and wheat growing practices at the farm and plot level. Furthermore, we assessed the household head’s level of risk aversion using a set of self-assessment and hypothetical yield scenario questions. In addition to the household survey data, GPS positions of ZT service providers who were active in the area were available from CSISA. The geographic coordinates of service providers were integrated into our modeling approach as an additional potential determinant of ZT adoption.

## Methodological approach

### Model estimation strategy

#### Accounting for non-exposure to the ZT technology

Following the approach of van de Ven and van Praag (1981)), we applied a two-stage estimation framework using a probit model with sample selection. The first-stage probit model identifies determinants of technology awareness; in the second stage, a probit model identifies determinants of technology adoption among the aware sub-sample. Since the selection process is non-random, non-exposure bias needs to be controlled for. This was achieved by including the Inverse Mills Ratio (IMR) in the second-stage probit model, analogous to the method proposed by Heckman (1979) for the case of a second-stage regression with a continuous dependent variable. The estimation approach of the probit model with sample selection (van de Ven and van Praag 1981) can be formalized as follows: let the adoption of ZT be based on a latent equation of the form

$$y_{i}^{*}=\beta x_{i}+u_{1i}$$ 1

where $y_{i}^{*}$ is the difference in expected returns between adopting and not adopting ZT, *X*_i_ is a vector of exogenous regressors, *β* is a vector of parameters to be estimated, and *u*_1_*_i_* is an error term. However, $y_{i}^{*}$ is not directly observable. Using idiosyncratic criteria, each household *i* will decide to adopt ZT if $y_{i}^{*}$ > 0, as reflected by the following equation with a binary observable outcome:

$$y_{i}=\beta X_{i}+u_{1i}$$ 2

where *y_i_* = 1 if $y_{i}^{*}$ > 0, i.e., the household uses ZT, and *y_i_* = 0 otherwise. As pointed out by Feder et al. (1985), a binary yes/no specification of the outcome variable has severe shortcomings in instances where there is great variation in the adoption *intensity*; i.e., a binary variable would not differentiate between farmers who used a new technology on their entire cultivated area and those who tested it on a small portion of their land. However, in the case of ZT wheat we find that, once the decision is made to use ZT, the practice is used on the entire wheat area by more than 82% of adopters (cf. section “Scale of zero-tillage wheat practice and development over time”), justifying the use of a binary dependent variable.

However, whether a household has a chance to decide whether or not to use ZT hinges on a selection equation of the form

$$a_{i}=\gamma Z_{i}+u_{2i}$$ 3

where *a_i_* takes on the value of 1 if ‘the household head has heard of ZT and knows in theory how the technology works’ and the value of 0 otherwise. This definition is in line with what is termed ‘knowledge exposure’ by Kabunga et al. (2012), which goes beyond mere ‘awareness exposure’, i.e. having heard of a technology. It implies that the farmer understands the attributes of the technology, which is of particular importance for knowledge-intensive innovations, such as conservation agriculture (Kabunga et al. (2012). Hence, *y_i_* is meaningfully observed only if *a_i_* = 1, since, otherwise, it will be 0 by definition. Furthermore, *u*_1_*_i_* and *u*_2_*_i_* are assumed to be distributed according to a bivariate Normal distribution with mean zero, standard deviation σ and correlation ρ. When ρ ≠ 0, standard probit techniques applied to Eq. 2 will yield biased estimates. To correct for the bias, consider that Prob (*a_i_* = 1) = Prob (*u*_2_*_i_* > − *γZ_i_*) = Prob (*γZ_i_*) = Φ (*γZ_i_*), where Φ is the cumulative distribution at *γZ_i_*. Then

$$E\left[y_{i}|a_{i}=1\right]=\beta X_{i}+\rho \sigma \lambda_{i}\left(\gamma Z_{i}\right)$$ 4

where λi = ϕ(γZi)/Φ(γZi), where ϕ is the probability density function at γZ_i_. λ_i_ is the IMR, and ρσ equals the regression coefficient on the IMR, *βλ*. As shown above, the IMR is the ratio of the value of the density function of a standard normal distribution evaluated at *γZ_i_* and the probability of being in the ZT-aware sub-sample, which equals the value of the cumulative distribution at *γZ_i_* for ZT-aware households and its complement to 1 for non-aware households. By including the IMR as an additional explanatory variable into the second-stage equation, potential non-exposure bias is corrected for:

$$y_{i}=\beta X_{i}+\beta_{\lambda }\lambda_{i}+u_{1i}$$ 5

We used the Stata 13 software package (www.stata.com) to estimate the probit model with sample selection (‘heckprobit’ model), specifying heteroskedasticity-consistent standard errors that account for clustering of the sample at the village level. The model produces a Wald test on the null hypothesis that ρ = 0,^4^ in which case non-exposure bias would not exist, and Eq. 5 would simplify to Eq. 2.

#### Accounting for social network effects in the adoption process

Since the seminal paper by Feder et al. (1985) on the adoption of agricultural innovations, which considered individualspecific farm and farmer characteristics as potential adoption determinants, micro-level adoption studies have been extended to include more dynamic elements related to social learning, i.e., learning about a new technology through the interaction with others (Foster and Rosenzweig 1995; Granovetter 2005; Feder and Savastano 2006). Hereby, empirical adoption studies have typically estimated an adoption equation of the form

$$y_{iv}=\beta X_{iv}+\alpha {\bar{y}}_{v}+kG_{v}+u_{iv}$$ 6

where *ӯ*_v_ denotes the village-level adoption rate as a proxy for measuring network effects and *G_v_* is a vector of other village-level regressors. Eq. 6 implies that an individual’s adoption decision is influenced by the adoption behavior of all other farmers in the village. However, this approach has been contested by studies showing that for gathering information and making adoption decisions farmers rather rely on small individual social networks which are not necessarily limited to village boundaries (Conley and Udry 2001; Bandiera and Rasul 2006; Matuschke and Qaim 2009). Given the social stratification by caste, such individual social networks may be of particular relevance in rural India (Matuschke and Qaim 2009). Hereby, farmers may not only be influenced by the adoption behavior of their individual social networks (endogenous network effect), but also by their network members’ characteristics, such as age, education, and caste (exogenous network effect), as differentiated by Manski (2000). Drawing on the approach of Matuschke and Qaim (2009), we account for endogenous and exogenous individual network effects as in the following equation:

$$y_{iv}=\beta X_{iv}+\alpha {\bar{y}}_{v}+\delta y_{n\left(i\right)}+\varepsilon X_{n\left(i\right)}+kG_{v}+u_{iv}$$ 7

where *y_n(i)_* denotes the adoption behavior of household *i*’s individual social network and *X_n(i)_* is a vector of exogenous network member characteristics. However, we extend the approach of Matuschke and Qaim (2009) by accounting for potential non-exposure bias as elaborated above. Hence, we estimate the following model:

$$y_{iv}=\beta X_{iv}+\beta_{\lambda }\lambda_{iv}+u_{1iv}$$ 8

where *X_iv_* encompasses all regressors included in Eq. 7 and *λ_iv_* is the IMR derived from an exposure equation of the form

$$a_{iv}=\dot{\gamma }{\dot{Z}}_{iv}+u_{2iv}$$ 9

where *Ż_iv_*, in addition to the vector of exogenous regressors from Eq. 3, contains the village-level adoption rate, endogenous and exogenous individual social network characteristics, and village-level characteristics as specified in Eq. 7.

### Model specification

The dataset encompassed 990 households with no missing values in any of the variables required for the analysis. Out of these, 439 respondents (44.3%) were aware of the ZT technology (according to our ‘knowledge-exposure’ definition given above), highlighting the need to account for potential non-exposure bias, as elaborated above.

According to Feder et al. (1985), farm size, risk exposure, human capital, labor availability, credit access, tenure security, and access to commodity markets are important determinants of the adoption of agricultural innovations. Based on their review and drawing on the concept of livelihood resources as laid out in the sustainable livelihoods framework (Chambers and Conway 1992; Scoones 1998), we hypothesize the adoption of ZT in wheat to be determined by the households’ asset base and risk preferences. The asset base includes access to relevant services and commodity markets and is subsumed under five types of capital, namely (1) natural capital, (2) human capital, (3) financial capital, (4) social capital/information access, and (5) market access/infrastructure. Table 1 provides the definitions and summary statistics of the dependent and explanatory variables used in the regression models. It further shows that the first-stage equation contains two dummy explanatory variables, radio and TV ownership, which are omitted from the second-stage; with respect to information access, mass media channels are of particular importance for knowledge acquisition, whereas the adoption decision is influenced more by interpersonal communication channels (Rogers 2003). Apart from being conceptually consistent, having at least one variable in the vector of selection equation regressors (*Z_i_*) which is not included in the regressors of the second stage (*X_i_*) is highly desirable for econometric reasons. If *Z_i_* and *X_i_* are identical, the IMR can be highly correlated with the elements of *X_i_*, leading to inflated standard errors (Wooldridge 2006: 620). Conversely, *X_i_* should be a strict subset of *Z_i_*, i.e. all explanatory variables in the second-stage equation are included in the selection equation to ensure consistent estimates (Wooldridge 2006).

**Table 1:** Definitions and summary statistics of dependent and explanatory variables in regression models explaining awareness and adoption of zero-tillage (ZT) technology in wheat in Bihar, India

Variable description		Awareness Stage (*N* = 9901)		Adoption conditionalon awareness (*N* = 439)	
		Mean.	Std. Dev	Mean	Std. Dev.
Dependent variables					
ZT awareness	= Dummy, = 1 if HH^2^ head at least knows about ZT in theory, 0 otherwise	0.443	0.497	-	-
ZT adoption	= Dummy, = 1 if HH used ZT in wheat in the 2012/13 rabi season, 0 otherwise	-	-	0.677	0.468
Natural capital					
Cultivable area	= Total area available for cultivation (ha)	1.276	1.312	1.685	1.455
Maximum plot size	= Size of largest irrigable plot (ha)	0.617	0.738	0.776	0.780
Land owned	= Dummy, = 1 if HH head owns land, 0 otherwise	0.897	0.304	0.927	0.260
Human capital					
Labor/land ratio	= Labor-to-land ratio (number of HH members aged 15 to 65 ha^−1^)	8.961	15.142	5.754	8.649
Age	= Age of HH head (years)	49.407	13.194	48.704	12.602
High education	= Dummy, = 1 if educational achievement of HH head is >12th grade, 0 otherwise	0.112	0.316	0.178	0.383
Low caste	= Dummy = 1 if HH belongs to Scheduled Tribe (ST) or Scheduled Caste (SC), 0 otherwise	0.124	0.330	0.098	0.298
High caste	= Dummy = 1 if HH belongs to General caste, 0 otherwise	0.403	0.491	0.474	0.500
Risk preference	= HH head’s general risk preference, self-assessed on a scale from 0 (= fully avoiding risk) to 10 (= fully prepared to take risk)	5.162	2.165	5.626	2.280
Financial capital					
Credit access	= Logged max. amount HH could currently borrow (‘000 INR)^3^	115.914	243.601	149.504	266.367
Information access					
Farmer association	= Dummy,= 1 if HH head is member of the local farmer association	0.020	0.141	0.025	0.156
Extension access	= Access to agricultural extension on a scale from 0 (= no access) to5 (= very good access)	2.606	1.382	2.847	1.402
Mobile phone	= Dummy,= 1 if HH owns at least one mobile phone, 0 otherwise	0.940	0.237	0.966	0.182
Radio	= Dummy,= 1 if HH owns at least one radio, 0 otherwise	0.239	0.427	-	-
TV	= Dummy,= 1 if HH owns at least one TV set, 0 otherwise	0.236	0.425	-	-
Social network characteristics					
NM ZT use^4^	= Share of network members (NMs) who used ZT earlier than respondent (%)	36.111	43.262	47.760	44.618
NM ZT use*smallest	= NM ZT use, interacted with smallest farm size tercile dummy variable	7.963	24.557	8.542	25.703
NM ZT use*middle	= NM ZT use, interacted with middle farm size tercile dummy variable	12.896	31.476	15.566	34.411
NM ZT use*largest	= NM ZT use, interacted with largest farm size tercile dummy variable	15.253	33.472	23.652	39.441
NM meet frequency	= Average monthly number of contacts with NMs	9.224	6.449	9.463	6.412
NM age	= Average age of NMs (years)	55.971	10.212	55.524	10.143
NM education	= Total number of years of education of NMs	21.172	12.142	24.831	12.326
NM higher caste	= Dummy, = 1 if all NMs belong to a higher caste than the respondent, 0 otherwise	0.101	0.301	0.098	0.298
Number of NMs	= Number of NMs provided by respondent	2.803	0.576	2.761	0.619
Village characteristics and infrastructure					
Village ZT use^4^	= Village-level ZT adoption rate (%)	28.722	27.591	32.057	27.916
Vill. ZT use*smallest	Village ZT use, interacted with smallest farm size tercile dummy variable	10.193	22.487	8.166	21.068
Vill. ZT use*middle	Village ZT use, interacted with middle farm size tercile dummy variable	9.066	19.314	10.053	20.872
Vill. ZT use*largest	Village ZT use, interacted with largest farm size tercile dummy variable	9.463	20.795	13.838	23.870
No ZT SP in 5 km	= Dummy, = 1 if there is no ZT service provider in 5 km radius, 0 otherwise	0.025	0.157	0.021	0.142
Dist. all-weather road	= Distance to all-weather road from village center (km)	0.716	1.088	0.790	1.057
Dist. input market	= Distance to local input market from village center (km)	4.217	3.525	4.382	3.666
Dist. output market	= Distance to output market from village center (km)	2.685	3.126	2.894	3.310
Late onset of rain	= Dummy, = 1 if monsoon rains occurred late within last 10 years, 0 otherwise	0.923	0.266	0.907	0.291
District dummies (Bhojpur is base district)					
Begusarai	= Dummy, = 1 if HH is located in Begusarai district, 0 otherwise	0.074	0.261	0.059	0.236
Bhojpur	= Dummy, = 1 if HH is located in Bhojpur district, 0 otherwise	0.477	0.500	0.542	0.499
Buxar	= Dummy, = 1 if HH is located in Buxar district, 0 otherwise	0.148	0.356	0.125	0.331
Lakhisarai	= Dummy, = 1 if HH is located in Lakhisarai district, 0 otherwise	0.075	0.263	0.086	0.282
Samastipur	= Dummy, = 1 if HH is located in Samastipur district, 0 otherwise	0.152	0.359	0.112	0.315
Vaishali	= Dummy, = 1 if HH is located in Vaishali district, 0 otherwise	0.075	0.263	0.075	0.264

1 10 cases with extreme values of the explanatory variable “Cultivated area” were excluded from the analysis since the data seemed unreliable; the extreme values exceeded the median of the variable by more than 5 standard deviations.

2 HH = Household.

3 For ease of interpretation, summary statistics are provided for the unlogged variable.

4 Summary statistics shown for illustration and ease of interpretation; the variable enters the model interacted with farm size tercile dummies only.

To be consistent with the notion of information *access*, we measured access to agricultural extension on a Likert scale indicating the extent to which information from the extension service was generally available to the respondent, rather than eliciting the number of extension visits received or field days attended, which measure both access to extension and whether the farmer chose to take advantage of it (Doss 2006). Along similar lines, we follow the approach of Diagne et al. (2000) to measure credit access by eliciting the amount that respondents *could* borrow from various formal and informal sources, rather than the amount actually borrowed, which confounds access to credit with demand for credit. In contrast to most models of technology adoption that treat risk preferences as an unobservable factor, we include a proxy variable of the household head’s risk preferences as an explanatory variable. The variable *Risk preference* uses a self-assessment scale and is based on the German Socio-Economic Panel Study (Gloede et al. 2011).

As elaborated in the previous section, drawing on the approach of Matuschke and Qaim (2009), a salient feature of our model with respect to the social capital base is the inclusion of the respondents’ individual agricultural information network characteristics as explanatory variables in both the awareness and adoption stages of our model. To this end, survey respondents were asked to provide some basic information on those three farmers whom they interacted with most frequently about agricultural issues, termed network members (NMs) in the following. To capture endogenous network effects, we collected data on the NMs’ ZT adoption status and, for adopters, the first year of use of the practice. The latter piece of information is crucial to address what Manski (1993) coined the reflection problem: while the behavior of the group (i.e., NMs) potentially influences the individual (i.e., the respondent), the reverse is also true. As suggested by Manski (2000), we therefore assume that the individual is influenced by the group with a time lag, and thus decides whether or not to adopt ZT depending on the NMs’ success with the technology. This ‘seeing is believing’ type of behavior has been documented in various empirical studies (e.g. Foster and Rosenzweig 1995; Dong and Saha 1998). Hence, only those NMs who used ZTearlier than the respondent enter our model as ZTadopters; those who adopted ZTin the same year or later than the respondent are considered non-adopters to avert the reflection problem. Another variable related to potential endogenous network effects measures the average frequency at which NMs and respondents meet. To capture potential exogenous network effects, i.e., those caused by who the NMs are, rather than how they behave, we include variables related to NMs’ age, education, and caste. Furthermore, since not every respondent provided information on threeNMs, the number of NMs is included in the model as a control variable. To some extent, the inclusion of information on NM characteristics may mitigate the econometric problem that peer group membership itself is likely to be endogenous (Matuschke and Qaim 2009; Songsermsawas et al. 2016) since individual social networks tend to be characterized by a high degree of homophily, i.e., they are usually formed among farmers of a similar social status (Rogers 2003).Amethodologically preferable approach would be the use of instrumental variables to control for potential endogeneity. Songsermsawas et al. (2016) used the characteristics of friends of the respondents’ network peers (who are unknown to the respondents themselves) as instruments for the peers’ characteristics, but such costly-to-collect data were not available in our case.

As farmers of different socioeconomic status are likely to have differential access to formal information sources about agricultural innovations (see section “Farmers’ individual agricultural information networks” below), the information gained through personal communication with their self-reported peers or by observing the behavior of other farmers in their village may influence their adoption decisions to varying extents.

Thus, other than previous empirical studies, we therefore disaggregated our estimated endogenous network effects by farm size tercile, i.e. we interacted individual-network and village-level ZT adoption rates with dummy variables indicating the farm size tercile the respondent household belonged to.

Since farmers’ access to the ZT technology largely depends on service providers (Erenstein and Laxmi 2008; Keil et al. 2016), a variable reflecting the proximity of a ZT service provider is included in the model. Using the GPS co-ordinates of ZT service providers in the survey districts, which were available through CSISA, we created variables measuring the number of ZT SPs in various radiuses around individual sample households, ranging from 500 m to 20 km. Based on the explanatory power of the different variables tested, a dummy variable indicating the absence of any ZTservice provider in a 5 km radius enters the final model. Furthermore, district dummies control for location-specific differences, which may be caused, among others, by varying timings and intensities of CSISA activities.

We estimated regression models of varying degrees of complexity in order to discuss their respective (de)merits, especially with respect to potential non-exposure- and endogeneity biases: Model 1 is a simple probit model, which neither accounts for non-exposure nor for potential individual social network effects (as Eq. 2 above); Model 2 is a probit model which includes individual social network characteristics; Model 3 uses the ‘heckprobit’ specification, hence correcting for potential nonexposure bias, but it omits individual network characteristics; finally, Model 4 accounts for both non-exposure bias and potential individual social network effects, corresponding to Eq. 8 above. Regression results are presented in Tables 5 and 6 in the section “Factors affecting knowledge of zero-tillage and adoption of the technology”. Table 1 lists the definitions and summary statistics of the dependent and explanatory variables used in the regression models.

## Results

### Zero-tillage wheat adoption and farmers’ awareness of the technology

Based on the census data collected across the surveyed villages we calculated the average ZT adoption rate by individual farm households at 27.5%, whereas village-level adoption rates ranged from 1% to 93%. To examine potential scale biases in awareness and use of ZT, the upper part of Table 2 displays basic farm characteristics and information on farmers’ awareness and use of the practice differentiated by farm size terciles, i.e., for the smallest, middle, and largest third of the sample households. Farmers in the smallest tercile had on average 0.3 ha available for farming, of which 90.8% were used for growing wheat during the rabi season. At 0.9 ha and 2.7 ha, average farm sizes were considerably larger in the middle and highest terciles, respectively (Column 1). At the same time, the area share under wheat was significantly smaller in these strata (Column 2). Plot size may be a limiting factor for the adoption of ZT which requires the use of a tractor-drawn seed drill. Since wheat is grown exclusively under irrigated conditions, the size of the largest available irrigable plot is displayed. Unsurprisingly, there are highly significant differences among farm size terciles, with, at 0.2 ha, the largest irrigable plot of farmers in the smallest tercile measuring only one sixth of that of the largest-tercile farmers, on average (Column 3).

Table 2 further illustrates highly significant differences with respect to educational achievement and caste membership, with the farmers with less land being less educated and belonging to lower castes, and vice versa (Columns 4–7). Furthermore, the table shows that, overall in the 2012/13 *rabi* season, 52.1% of households had heard of ZT (i.e., they were aware), 44.3% knew how the technology works (they were aware and knowledgeable), and 30.0% used the practice (Columns 8–10). This means that 57.6% of aware households and 67.7% of knowledgeable households chose to use the technology. Looking at farm size terciles, we found a substantial and statistically highly significant scale bias between farm size terciles with respect to awareness (1:1.6:2.2 ratio among terciles; Column 8) and knowledge (1:1.5:2.4; Column 9). However, across landholding terciles, the share of knowledgeable households that chose to use the technology was very similar at 68.9% among the smallest-, 68.3% among the middle-, and 66.7% among the largest-tercile farmers.

**Table 2:** Basic farm characteristics, zero-tillage (ZT) related awareness and knowledge, and use of ZT among sample households (HHs) in the 2012/13 rabi season, differentiated by farm size terciles (values are means apart from columns 5–10)

RespondentCharacteristics	(1)Cultivable area (ha)	(2)Wheat area share (%)	(3)Size of largest irrigable plot (ha)	(4)Years of education of HH head	(5)% HH heads with education >12th grade)	(6)% HH heads belonging to Scheduled castes	(7)% HH heads belonging to General caste	(8)% HHs having heard of ZT	(9)%HHs knowing how ZT works	(10)%HHs using ZT	(11)Predicted prob. of ZT adoption (%)^1^
Farm size tercile											
Smallest (N = 330)	0.28^a^	90.83^a^	0.20^a^	5.52^a^	4.24	22.73	23.94	32.73	27.27	18.79	17.91^a^
Middle (N = 338)	0.89^b^	77.20^b^	0.47^b^	7.54^b^	10.36	10.06	41.72	52.96	42.01	28.70	28.71^b^
Largest (N = 322)	2.70^c^	68.38^c^	1.21^c^	9.36^c^	19.25	4.35	55.59	71.12	64.29	42.86	43.60c
Stat. sig.	****^2^	****^2^	****^2^	****^2^	****^3^	****^3^	****^3^	****^3^	****^3^	****^3^	****^2^
ZT adoption status											
Adopters (N = 297)	1.69	76.83	0.81	9.10	19.19	7.41	50.84	100.00	100.00	100.00	44.37
Non-adopters (N = 693)	1.10	79.76	0.53	6.75	7.79	14.57	35.79	31.60	20.49	0.00	23.77
Stat. sig.	****^2^	*^2^	****^2^	****^2^	****^3^	***^3^	****^3^	****^3^	****^3^	By def.	****^2^
Whole sample (N = 990)	1.28	78.88	0.62	7.46	11.21	12.42	40.30	52.12	44.34	30.00	29.95

*(**)[***]{****} Significant at the 10%(5%)[1%]{0.1%} level of alpha error probability.

1 Based on Model 4.

2 Based on (multiple) Mann-Whitney tests, accounting for family-wise error; diverging superscript letters indicate statistical significance at least at the indicated level.

3 Based on chi-square test.

The lower part of Table 2 provides a comparison of adopters and non-adopters of ZT and shows that, on average, adopters had significantly larger farms and plots and were better educated and of higher caste status than non-adopters (Columns 1–7). Despite the fact that the survey focused on villages with some level of ZTadoption (cf. section BResearch area, sampling procedure, and data collection”), only 31.6% of non-users had ever heard about the technology (Column 8) and 20.5% knew in theory how it worked (Column 9). As elaborated in the previous section, our analysis of adoption determinants (Eq. 8) is based on the knowledgeable sub-sample composed of 439 households (44.3% of samplehouseholds).

### Scale of zero-tillage wheat practice and development over time

Table 3 examines the scale of use of the ZT practice among adopters from 2010, the first full year of operation of CSISA, to 2012. Over this period, the number of ZT users in the study villages grew by 47.0% from 202 to 297. In the 2012/13 rabi season, the average adopter grew approximately 1.1 ha of ZT wheat, representing 89.4% of their total wheat area; 82.5% of adopters did not grow any wheat with conventional tillage, i.e., their adoption scale amounted to 100% (‘full’ adoption as opposed to ‘partial’ adoption). Among the 202 ZT users in the 2010/11 rabi season, the average ZT wheat area amounted to 1.2 ha, equivalent to 95.1% of the farmers’ total wheat area, and the share of full adopters was 90.6%. Differentiating by farm size terciles, the absolute area under ZT wheat differed among terciles due to their different land endowment. However, in 2010, the area share under ZT wheat did not differ significantly among terciles, and neither did the share of full adopters.With respect to the development of the social inclusiveness of ZT use over time, it is interesting to note that the number of ZT adopters in the smallest landholding tercile grew by 77.1% between 2010 and 2012, whereas the growth rate was lower at 47.0 and 36.6% among the middle- and largest-landholding terciles, respectively. Furthermore, the average ZT share among the smallest- and middle-tercile landholders became significantly larger (94.0%) than that of adopters in the largest tercile (84.3%).

The bottom part of Table 3 shows the development of the scale of ZT adoption over time among those 202 farmers who had used the technology already in the 2010/11 *rabi* season. We found that 196 farmers (97%) had at least maintained their (very high) 2010 share of ZT wheat in the 2012/13 growing season, with no significant difference among landholding terciles. The remaining six households had reduced their ZT wheat share by up to 30%, leading to a slight overall annual reduction by 0.24%, on average. The large share of full adopters among ZT users in both seasons and the high level of adoption stability over time justify the specification of the dependent variable in the adoption stage of the regression model as a dichotomous variable, indicating either use or non-use of the technology.

**Table 3:** Scale of zero-tillage (ZT) use among adopters and development over time, differentiated by farm size terciles

	Whole sample	Smallest tercile	Middle tercile	Largest tercile	Sig. of diff.
*Rabi* season 2012/13	(*N* = 297)	(*N* = 62)	(*N* = 97)	(*N* = 138)	
Mean cultivable area (ha)	1.69	0.32^a^	0.94^b^	2.82^c^	***^1^
Mean ZT wheat area (ha)	1.05	0.28^a^	0.79^b^	1.59^c^	***^^1^^
Mean ZT share within total wheat area (%)	89.38	93.96^a^	93.69a	84.29b	*^1^
Share of households using ZT wheat only (%)	82.49	91.94	87.63	74.64	**^2^
Rabi season 2010/11	(*N* = 202)	(*N* = 35)	(*N* = 66)	(*N* = 101)	
Mean cultivable area (ha)	1.82	0.35^a^	0.96^b^	2.89^c^	***^1^
Mean ZT wheat area (ha)	1.23	0.30^a^	0.81^b^	1.83^c^	***^1^
Mean ZT share within total wheat area (%)	95.12	93.41	98.18	93.71	n.s.^1^
Share of households using ZT wheat only (%)	90.59	88.57	95.45	88.12	n.s.^2^
Development of the scale of ZT use over time^3^	(*N* = 202)	(*N* = 35)	(*N* = 66)	(*N* = 101)	
Average annual change of ZT wheat share from 2010/11 to 2012/13 (%)	−0.24	0.51	−0.65	−0.23	n.s.^1^
Share of households at least maintaining the 2010/11 ZT	97.03	100.00	96.97	96.04	n.s.^2^
wheat share in 2012/13 (%)					

Note: Terciles are based on the whole sample as in Table 2, not on the sub-sample of ZT users.

*(**)[***] Statistically significant at the 5% (1%)[0.1%] level of alpha error probability.

1 Based on multiple Mann-Whitney tests, accounting for family-wise error.

2 Based on Chi-square test.

3 Based on farmers who had used ZT in the 2010/11 Rabi season.

### Farmers’ stated motivating factors for zero-tillage adoption and evaluations of the practice

As primary^5^ negative perceptions of conventional-tillage (CT) wheat, respondents cited (1) high cost of fuel for soil preparation (43.6% of respondents), (2) labor shortage for soil preparation (19.2%), and (3) that soil preparation equipment or services were not always available on time (10.6%; multiple responses possible). While there was no significant difference between farm size classes regarding the frequency with which these concerns were cited, the severity of all three concerns was rated significantly higher by farmers in the largest landholding tercile as compared to those in the smallest tercile.^6^

Not surprisingly, the primary reasons stated for shifting from CT wheat to ZT wheat were (1) reduced cost (46.2% of ZT adopters) and (2) reduced labor requirements (22.5%; multiple responses possible). The most widespread concerns with ZT wheat were (1) that ZT services were not always available on time (19.7% of ZTadopters), (2) high cost of the ZT drill (9.8%), (3) yield reduction due to increased weed infestation (9.3%), (4) high cost of ZT services (7.4%), and (5) increased expenses for herbicides (6.6%; multiple responses possible). Forty-six percent of ZT adopters did not cite any problem with the technology.

Overall, ZT adopters reported a high degree of satisfaction with the technology, assigning an average satisfaction score of 4.2 (on a scale from −5 (= very dissatisfied) to +5 (= very satisfied)) to the practice. The mean satisfaction score among adopters in the smallest farm size tercile (3.8) was slightly lower than that in the medium (4.2) and largest (4.4) terciles (Mann-Whitney tests significant at P<0.01 and P < 0.001, respectively). satisfied)) to the practice. The mean satisfaction score among adopters in the smallest farm size tercile (3.8) was slightly lower than that in the medium (4.2) and largest (4.4) terciles (Mann- Whitney tests significant at *P*<0.01 and *P* < 0.001, respectively).

### Farmers’ individual agricultural information networks

Among the knowledgeable sub-sample of 439 sample farmers, 73.1% cited ‘fellow farmers’ as their primary information source about the technology, supporting our hypothesis that farmers’ individual information networks play an important role in the diffusion of ZT technology. Furthermore, we found that the share of farmers citing this informal information source differed significantly among farm size terciles, amounting to 90.0%, 71.8%, and 66.7% among the smallest, middle, and largest tercile respondents, respectively (chisquare test significant at *P* < 0.001). The high importance of peer networks especially for the smallest farmers is corroborated by our finding that only 24.4% of the smallest-tercile farmers accessed more formal sources of agriculture related information (such as farmers’ fairs, input dealers, and the agricultural extension service), whereas 38.7% and 55.6% among the middle- and largest-tercile farmers did so, respectively (chi-square test significant at *P* < 0.001). These findings emphasize that social network effects may differ between socioeconomic strata, warranting the estimation of disaggregated coefficients in the subsequent regression analysis.

In the following, we describe basic characteristics of our respondents’ individual social networks and explore how these characteristics varied between socioeconomic strata, with farm size terciles and ZT adoption status as proxies (Table 4). Column 1 shows that there was no difference in the number of NMs cited by respondents in the different groups (2.8, on average). Column 2 displays the farm sizes of the NM groups. Among respondents in the smallest tercile, the mean farm size of NMs amounted to 1.3 ha, it was 1.9 ha among respondents in the middle tercile, and 3.0 ha among those in the largest tercile; all means are statistically different from each other at *P* < 0.001. Since farm size is an important wealth indicator, this finding indicates that the respondents interacted mostly with farmers who were close to their own social stratum. However, comparing the mean farm size of NMs with that of the respondent farmers in the respective farm size tercile (Table 2, Column 1) reveals a clear tendency of an ‘upward orientation’ among the smallest- and middle-tercile respondents, i.e., they cited network members whose farms were approximately one hectare larger than their own, on the average (Wilcoxon signed-rank tests significant at *P* < 0.001); the observed average difference of 0.3 ha among the largest-tercile respondents is not statistically significant. Consistent with our finding that ZT adopters had larger farms than nonadopters (Table 2, Column 1), also the mean farm size of adopters’ NMs exceeded that of non-adopters’ NMs by a substantial 74%, on average (Table 4, Column 2).

**Table 4:** Major characteristics of respondents’ personal network members^1^ (NMs), differentiated by respondents’ farm size tercile and zero-tillage (ZT) adoption status (values are means)

Respondent characteristics	(1)No. of NMs cited	(2)Farm size of NMs(ha)^2^	(3)Age of NMs (years)	(4)Years of education of NMs	(5)No. of NMs belonging to Scheduled castes	(6)No. of NMs belonging to General caste	(7)% NMs residing in same village as resp.	(8)No. of times resp. meet NMs per month	(9)No. of ZT users among NMs	(10)No. of NM ZT users who had adopted earlier than respondent
Farm size tercile										
Smallest (N = 330)	2.79	1.25^a^	55.2	6.15^a^	0.47^a^	0.91^a^	90.6	10.67^a^	0.75^a^	0.66^a^
Middle (N = 338)	2.78	1.92^b^	55.8	7.78^b^	0.23^b^	1.30^b^	90.3	8.88^b^	1.15^b^	1.04^b^
Largest (N = 322)	2.84	3.01^c^	56.9	9.02^c^	0.09^c^	1.66^c^	92.0	8.12^b^	1.66^c^	1.36^c^
Stat. sig.	n.s.	****	n.s.	****	***	***	n.s.	***	****	***
ZT adoption status										
Adopters (N = 297)	2.81	2.95	55.2	9.14	0.16	1.52	90.7	9.44	1.95	1.41
Non-adopters (N = 693)	2.79	1.70	56.3	6.99	0.31	1.19	91.1	9.14	0.85	0.85
Stat. sig.	n.s.	****	*	****	***	****	n.s.	n.s.	****	****
Whole sample (N = 990)	2.80	2.08	56.0	7.64	0.26	1.29	91.0	9.23	1.18	1.02

*(**)[***]{****} Significant at the 10%(5%)[1%]{0.1%} level of alpha error probability, based on (multiple) Mann-Whitney tests, accounting for family-wise error; diverging superscript letters indicate statistical significance at least at the indicated level.

1 “Network members” refers to up to three farmers the respondent communicates with most about agricultural issues.

2 Since respondents could not always provide this information, this is based on 843 observations only. *N* = 268, 290 and 285 for the smallest, middle, and largest tercile, respectively; *N* = 253 for adopters and 590 for non-adopters.

While the respondents were, on average, 49.4 years old at the time of the survey (cf. Table 1), theNMs they cited as their primary contacts were on average 56 years old (Table 4, Column 3); the NMs of ZT adopters were slightly younger than those of non-adopters. With respect to educational achievement and caste membership, Columns 4–6 reveal similar patterns among NMs as among the survey respondents themselves (cf. Table 2, Columns 4–7); e.g., farmers in the smallest tercile who tended to be less educated than larger farmers, had a greater number of farmers with low formal education levels among their NMs, while the opposite was true for farmers in the largest tercile.

There was no difference between farm size terciles or ZT adopters and non-adopters in the high level of concentration of NMs in the respondents’ own village (91%, on average, Column 7), whereby respondents in the smallest farm size tercile met their NMs slightly more often than respondents with larger farms (Column 8).

Column 9 shows that the average number of ZT users among the respondents’ NMs differed significantly among farm size terciles, ranging from 0.75 among the smallest to 1.66 among the largest tercile; furthermore, ZT adopters had significantly more ZT users among their NMs (1.95) than nonadopters (0.85). Of particular relevance for our subsequent regression analysis is the number of ZT users who had adopted the technology earlier than the survey respondent and could, therefore, have influenced the respondent’s adoption decision (Column 10). We find similar differences as in Column 9, i.e. those with larger farmers and ZTadopters had a greater number of ZT users among their NMs than those with smaller farmers and non-adopters of ZT.

### Factors affecting knowledge of zero-tillage and adoption of the technology

Table 5 presents the parameter estimates for factors influencing the adoption of ZT without accounting for exposure to the technology, whereby Model 1 omits and Model 2 includes individual network characteristics. Table 6 presents the estimates produced by the heckprobit specification, differentiating factors influencing knowledge of ZT (Stage 1) and, conditional on being knowledgeable, factors influencing the adoption of the technology (Stage 2); Model 3 omits and Model 4 includes individual network characteristics. We tested the models for potential multicollinearity of explanatory variables by calculating Variance Inflation Factors (VIFs); we find that, apart from the VIFs on the variables *Cultivable area* and its squared term, which are high by definition, the maximum and mean values of VIFs in the adoption stage of Model 4 amount to 2.89 and 1.71, respectively, indicating no cause for concern with regard to multicollinearity. Myers (1990) suggests that a value of 10 should not be exceeded by individual VIFs. Due to a larger number of observations and/or fewer explanatory variables, VIF levels are lower in the awareness stage of Model 4 and in the other model specifications. The robustness of the estimates is corroborated by the fact that the exclusion of non-significant explanatory variables leads to only minor changes in the coefficients of the remaining factors and their statistical significance levels. The estimation of the standard errors accounts for the two-stage sampling procedure with 40 village-level clusters. The explanatory power of the models in terms of overall share of cases correctly predicted is very similar, ranging from 71.5% in Model 1 to 73.4% in Model 4 (see bottom rows of Tables 5 and 6). Differentiating cases of ZTadopters and non-adopters reveals that close to 90% of non-adopting cases are correctly predicted, whereas the share of correctly predicted adopter cases is substantially lower and ranges more widely among the models, from 29.6% in Model 1 to 38.1% in Model 4. Adopter cases are ‘correctly’ predicted if the predicted probability of ZT adoption is > = 50%. As shown in Column 11 of Table 2, based on Model 4, the mean predicted probability of adoption amounts to 44.4% among the ZT adopter sub-sample, whereas it averages only 23.8% among non-adopters; the difference is statistically highly significant at *P* < 0.001. Furthermore, the predicted probabilities of adoption overall and disaggregated by farm size tercile match the observed incidence of adoption exceptionally well, indicating that the model has substantial explanatory power. However, the level of predicted probabilities among the adopter subsample is too low to correctly classify the majority of cases as adopters based on the conventionally used cut-off probability of 50%. Despite the low level of correct predictions among the ZT adopters, the inclusion of individual network characteristics in Models 2 and 4 leads to an increase in the share of correctly predicted adopter cases by 7.1 percentage points as compared to Models 1 and 3. Furthermore, a Wald test of independence of equations applied to Models 3 and 4 confirms that the error terms of the first- and second-stage regressions are correlated (*P* < 0.01), hence justifying the use of the heckprobit model. Taken together, specification tests and explanatory power indicate that Model 4 is the preferred specification.

**Table 5:** Maximum Likelihood estimates of probit models explaining the adoption of zero-tillage (ZT) wheat in Bihar; coefficients are marginal effects

Variable	Model 1: Probit model		Model 2: Probit model	
	without individual network characteristics		with individual network characteristics	
	Coefficient^1^	z-value^2^	Coefficient^1^	z-value^2^
Cultivable area	0.1390	3.32^***^	0.1228	2.74^***^
Cultivable area, sqd.	−0.0191	−3.08^***^	−0.0178	−2.84^***^
Maximum plot size	−0.0068	−0.21	0.0133	0.40
Land Owned^d^	0.0996	1.74^*^	0.1025	2.04^**^
Labor/land ratio	−0.0027	−1.57	−0.0024	−1.67^*^
Age	−0.0020	−1.86^*^	−0.0014	−1.26
High education^d^	0.1188	2.28^**^	0.0801	1.51
Low caste^d^	−0.0341	−0.53	−0.0578	−0.96
High caste^d^	0.0916	1.92^*^	0.0873	2.02^**^
Risk aversion	0.0183	2.65^***^	0.0178	2.74^***^
Credit access	0.0040	1.01	0.0004	0.11
Farmer association^d^	0.1088	0.93	0.0955	0.81
Extension access	0.0165	1.94^*^	0.0226	2.45^**^
Mobile phone^d^	0.0618	0.94	0.0475	0.70
Radio^d^	0.0129	0.41	0.0010	0.03
TV^d^	0.0626	1.34	0.0591	1.34
NM ZT use*smallest	-		0.0017	2.49^**^
NM ZT use*middle	-		0.0012	1.72^*^
NM ZT use*largest	-		0.0007	1.28
NM meet frequency	-		0.0077	2.73^***^
NM age	-		−0.0028	−1.67^*^
NM education	-		0.0044	2.96^***^
NM higher caste^d^	-		0.0565	1.21
Number of NMs	-		−0.0476	−1.57
Vil. ZT use, smallest	0.0019	2.12^**^	0.0015	1.61
Vil. ZT use, middle	0.0014	1.59	0.0010	1.04
Vil. ZT use, largest	0.0018	2.18^**^	0.0019	2.28^**^
No ZT SP in 5 km^d^	−0.2031	−4.98^****^	−0.1849	−4.06^****^
Dist. all−weather road	−0.0030	−0.15	−0.0100	−0.63
Dist. input market	0.0062	0.93	0.0054	0.79
Dist. output market	−0.0064	−0.73	−0.0077	−0.88
Late onset of rain^d^	0.1106	3.61^****^	0.1042	4.61^****^
Begusarai^d^	−0.1539	−3.46^***^	−0.1270	−2.99^***^
Buxard	−0.0184	−0.44	−0.0519	−1.13
Lakhisarai^d^	−0.1905	−4.16^****^	−0.1770	−3.59^****^
Samastipur^d^	−0.1812	−3.21^***^	−0.2044	−4.20^****^
Vaishali^d^	−0.1985	−4.31^****^	−0.2053	−4.83^****^
Number of observations =		990		990
Log likelihood =		−513.83		−494.14
Pseudo R−squared =		0.150		0.183
Explanatory power:				
Cases of ZT adopters correctly predicted (%) =		29.6		36.7
Cases of ZT non−adopters correctly predicted (%) =		89.5		88.0
Overall cases correctly predicted (%) =		71.5		72.6

*(**)[***]{****} Significant at the 10%(5%)[1%]{0.1%} level of alpha error probability

1 Coefficients are marginal effects (evaluated at means of all explanatory variables); for dummy variables, marginal effects are for a discrete change from 0 to 1

2 Based on robust standard errors adjusted for 40 village−level clusters

d Dummy variable

**Table 6:** Maximum Likelihood estimates of Heckman probit selection model explaining awareness of zero-tillage (ZT; 1st stage) and adoption of ZT wheat conditional on awareness (2nd stage) in Bihar; coefficients are marginal effects

	Model 3: Probit selection model				Model 4: Probit selection model			
	without individual network characteristics				with individual network characteristics			
	Awareness		Adoption cond. on awareness		Awareness		Adoption cond. on awareness	
Variable^d^	Coefficient^1^	z-value^2^	Coefficient^1^	z-value^2^	Coefficient^1^	z-value^2^	Coefficient^1^	z-value^2^
Cultivable area	0.2184	4.86****	0.1316	3.45***	0.1859	4.63****	0.1167	2.73***
Cultivable area, sqd.	−0.0235	−3.81****	−0.0180	−3.27***	−0.0212	−3.87****	−0.0169	−2.92***
Maximum plot size	−0.0283	−1.01	0.0034	0.11	−0.0008	−0.03	0.0232	0.71
Land owned^d^	0.0676	1.37	0.1175	1.61	0.0413	0.85	0.1220	1.75*
Labor/land ratio	−0.0031	−1.98**	−0.0020	−1.22	−0.0030	−2.33**	−0.0013	−0.77
Age	−0.0021	−1.76*	−0.0015	−1.33	−0.0016	−1.34	−0.0010	−0.86
High education^d^	0.1465	3.66****	0.1074	2.57**	0.0909	2.26**	0.0761	1.68*
Low caste^d^	0.0092	0.13	−0.0401	−0.61	−0.0072	−0.11	−0.0729	−1.06
High caste^d^	0.0685	1.96*	0.1019	2.34**	0.0517	1.66*	0.1015	2.34**
Risk preference	0.0174	2.58**	0.0200	3.45***	0.0172	2.67***	0.0186	3.08***
Credit access	0.0090	2.68***	0.0026	0.59	0.0049	1.51	−0.0008	−0.18
Farmer association^d^	0.0133	0.13	0.0970	0.97	0.0069	0.08	0.0812	0.80
Extension access	0.0270	2.22**	0.0148	1.93*	0.0318	3.06***	0.0201	2.09**
Mobile phone^d^	0.0715	1.26	0.0664	0.86	0.0607	1.20	0.0620	0.75
Radio^d^	0.1224	4.45****	-		0.1026	4.19****	-	
TV^d^	−0.0410	−1.41	-		−0.0384	−1.24	-	
NM ZT use*smallest	-		-		0.0025	3.94****	0.0017	2.90***
NM ZT use*middle	-		-		0.0010	1.79*	0.0012	2.09**
NM ZT use*largest	-		-		0.0011	1.91*	0.0007	1.36
NM meet frequency	-		-		0.0081	3.61****	0.0066	2.65***
NM age	-		-		−0.0017	−1.12	−0.0017	−1.05
NM education	-		-		0.0062	4.55****	0.0033	2.15**
NM higher casted	-		-		−0.0092	−0.24	0.0605	1.24
Number of NMs	-		-		−0.1069	−3.76****	−0.0316	−0.95
Vill. ZT use*smallest	0.0019	1.96*	0.0022	2.52**	0.0012	1.24	0.0016	1.78*
Vill. ZT use*middle	0.0020	2.05**	0.0016	1.69*	0.0018	2.05**	0.0012	1.16
Vill. ZT use*largest	0.0001	0.06	0.0022	2.43**	0.0001	0.05	0.0023	2.31**
No ZT SP in 5 km^d^	0.0113	0.17	−0.2990	−3.47***	0.0873	1.24	−0.2646	−2.72***
Dist. all-weather rd.	0.0066	0.29	−0.0022	−0.12	−0.0010	−0.05	−0.0119	−0.84
Dist. input market	0.0031	0.46	0.0055	0.85	0.0010	0.15	0.0053	0.78
Dist. output market	−0.0037	−0.33	−0.0055	−0.67	−0.0047	−0.46	−0.0069	−0.86
Late onset of rain^d^	−0.0675	−1.09	0.1199	2.93***	−0.0499	−1.06	0.1145	3.13***
Begusarai^d^	−0.2504	−4.28****	−0.1375	−1.86*	−0.1971	−4.01****	−0.1162	−1.68*
Buxar^d^	−0.0744	−1.38	0.0078	0.15	−0.1122	−2.24**	0.0012	0.02
Lakhisarai^d^	−0.1673	−2.18**	−0.2601	−3.17***	−0.1336	−1.80*	−0.2425	−2.53**
Samastipur^d^	−0.0979	−1.37	−0.1963	−2.37**	−0.1108	−1.52	−0.2341	−2.86***
Vaishali^d^	−0.0854	−0.93	−0.2315	−2.98***	−0.0935	−1.20	−0.2513	−2.92***
N =	990		439		990		439	
Log pseudolikelihood =	−793.64				−754.90			
Wald test of independent equations: chi-square (1) =	10.35^***^				8.13***			
Explanatory power:								
Cases of ZT adopters correctly predicted (%) =	31.0				38.1			
Cases of ZT non-adopters correctly predicted (%) =	89.6				88.6			
Overall cases correctly predicted (%) =	72.0				73.4			

*(**)[***]{****} Significant at the 10%(5%)[1%]{0.1%} level of alpha error probability.

1 Coefficients are marginal effects (evaluated at means of all explanatory variables); for dummy variables, marginal effects are for a discrete change from 0 to 1.

2 Based on robust standard errors adjusted for 40 village-level clusters.

d Dummy variable.

The following interpretation of regression results is therefore based onModel 4, which differentiates between factors affecting awareness of ZT (Eq. 9) and those affecting the adoption of the technology conditional on awareness (Eq. 8). It accommodates both endogenous and exogenous network effects, whereby endogenous effects are disaggregated by farm size tercile, as elaborated in the section “Model specification”. Model 4 produces estimates that are consistent with those of Model 3 (which omits network characteristics), indicating that the bias caused by the introduction of potentially endogenous network characteristics is negligible. Divergences between the two models are explicitly mentioned. Furthermore, the additional insights gained as compared to the simpler Models 1 and 2, which do not account for non-exposure bias, are highlighted.

The variable *Cultivable area* is included in the models as a wealth indicator and a factor that may influence the adoption of ZT directly, as the provision of ZT services on small farms is associated with higher per-hectare transaction costs and, hence, is likely less attractive for service providers (Keil et al. 2016). The model indicates a statistically highly significant positive quadratic relationship both with respect to awareness of ZT (henceforth awareness stage) and adoption of the technology conditional on being aware (henceforth adoption stage), i.e., farmers with greater land resources are more likely to know about and adopt ZT, but the marginal effect decreases with increasing farm size. In addition to farm size, plot size may influence the adoption of ZT. Very small plots can pose a technical limit to operating four-wheel tractor based equipment; moreover, per-hectare transaction costs for ZT services increase with decreasing plot size, potentially influencing the willingness of service providers to cater to very small plots. We therefore include the variable *Max. plot size* in the model, which measures the size of the largest irrigable plot available.^7^ While this variable is relatively strongly correlated with *Cultivable area* (Pearson correlation coefficient 0.76), a VIF of 2.62 and the statistically highly significant regression coefficients on *Cultivable area* and its squared term indicate no cause for concern regarding multicollinearity. However, the marginal effect of *Max. plot size* is not statistically significant in any of the model specifications. While there is strong evidence of immediate benefits from the use of ZT in wheat in Bihar in terms of yield increase and cost savings (Keil et al. 2015), the dummy variable *Land owned* controls for land tenure status, since the potential long-term soil fertility enhancing effect of ZT, if used in conjunction with residue retention, may incentivize farmers to use the technology on land they own. The estimated marginal effect is weakly statistically significant in the adoption stage of Model 4, indicating that the propensity to use ZT increases by 12.2 percentage points if the land is owned rather than leased in. In Model 3, the estimate is slightly smaller and statistically significant with an error probability of 11% only.

With respect to human capital endowment, several factors are statistically significant in the awareness and adoption stages. The labor/land ratio has a negative effect on the awareness of ZT, indicating that households with relatively more abundant family labor are less likely to gather information about novel mechanized technologies, which are generally labor-saving. The effect in the adoption stage is not statistically significant, however. This is plausible, as the alternative to hiring ZT services is usually the hiring of plowing services, which has implications on the extent of hired labor use rather than use of family labor.^8^ The marginal effect of the age of the household head is negative across all model specifications, but weakly significant in the models without inclusion of NM characteristics only; hence, there is only a weak indication that older farmers are less informed about novel agricultural practices.^9^ Much more important is the influence of education, with *High education* increasing the likelihood of being aware of ZT by 9.1 percentage points and the likelihood of adopting (conditional on awareness) by 7.6 percentage points. There is evidence of a positive influence of belonging to the General caste (*High caste*) as compared to the base category of ‘Backward caste’ in both the awareness and adoption stages, while the influence of belonging to scheduled castes (*Low caste*) is not statistically significant. Based on our self-assessment measure of *Risk preference*,we find strong evidence that farmers who are less risk averse are significantly more likely to be aware of ZT, and to adopt the technology once they are aware.

Since most farmers access ZT technology through custom hire services rather than their own machinery, financial capital, as proxied by *Credit access*, is not expected to play a significant role in the adoption process. Indeed, the null-hypothesis cannot be rejected in (the adoption stages of) all model specifications. However, in Model 3, the variable has a significant positive influence in the awareness stage, indicating that households with greater access to credit aremore likely to seek information about new technologies. Accounting for potential individual social network effects in Model 4 renders the variable significant with an error probability of 14% only.

The prerequisite to seeking information is having information access; both Models 3 and 4 show that access to agricultural extension positively influences farmers’ awareness and adoption of ZT, whereby the marginal effect is larger in the awareness stage. Furthermore, both models estimate similar and highly significant positive effects of radio ownership on the likelihood of knowing about ZT, at 12.2 and 10.2 percentage points, respectively. It is important to note that the simpler Models 1 and 2 fail to detect the relevance of radio ownership for accessing information about agricultural innovations.

The salient feature of Models 2 and 4 is the inclusion of the respondents’ individual network characteristics. Both models indicate that the NMs’ ZT adoption positively influences the adoption behavior of farmers in the smallest and middle farm size terciles (NM ZT use*smallest and NM ZT use*middle); a one-percentage point increase in the ZT adoption rate among the smallest (middle) farmers’ NMs entails a 0.17 (0.12) percentage point increase in their propensity to adopt the technology themselves. For the largest-tercile farmers, the estimated coefficient is also positive, but not significantly different from zero. Model 4, in addition, shows that NMs’ adoption of ZT significantly influences knowledge of the technology among farmers of all terciles, whereby the marginal effect is particularly large (0.25 percentage points) and statistically highly significant among the smallest tercile.

With respect to potential exogenous network effects, the respondent’s average number of contacts with the NMs (*NM meet frequency*), which we use as a proxy for the intensity of contacts within the network, has a statistically highly significant influence both in the awareness and in the adoption stage. Furthermore, the model provides strong evidence that the NMs’ level of education affects both knowledge and adoption of ZT, whereby the effect is particularly pronounced in the awareness stage. We further account for a potential caste related effect (*NM higher caste*) which is not found to be statistically significant. Finally, since 115 respondents (11.6%) provided data on less than three NMs, we include the number of NMs as a control variable.

All models account for potential village-level network effects by including the village-level ZT adoption rate, whereby again we allow the effects to vary between farm size terciles. Across all model specifications, we find a statistically significant positive influence on ZT adoption among the largest tercile (Vill. ZT use*largest), whereby Models 1 and 2, which do not correct for non-exposure bias, produce somewhat smaller marginal effects than Models 3 and 4. Omitting individual network characteristics, Models 1 and 3 show statistically significant village-level network effects on ZT adoption by the smallest-tercile farmers as well. The effect on middle-tercile farmers is weakly significant in Model 3 only. In the first stage, Models 3 and 4 produce a statistically significant positive effect of the village-level adoption rate on ZT awareness among farmers in the middle tercile, whereas it has clearly no effect on the largest tercile farmers. Omitting individual network characteristics, Model 3 estimates a significant village-level effect on technology awareness among the smallest tercile as well, whereas Model 4 attributes the effect to farmers’ individual networks, rendering the village-level coefficient statistically insignificant.

A number of variables control for other potential village-level effects, which are found to mostly influence the adoption stage. Across all models, the variables related to road and market infrastructure are statistically insignificant with very small z-values. However, two other factors are significant across all four model specifications: since for most farmers the use of ZT hinges on access to custom-hire services, in villageswhere there is no ZT service provider within a 5 km radius the likelihood of adopting the technology is estimated to decline by 29.9 and 26.5 percentage points in Models 3 and 4, respectively. Not accounting for exposure bias, Models 1 and 2 produce somewhat smaller marginal effects. Furthermore, we find strong evidence that the farmers’ decision to adopt is also influenced by climatic conditions. In villages that witnessed a delayed onset of monsoon rains in the past 10 years, the propensity to use ZT was approximately 11.5 percentage points higher than in villages where such delays were not experienced.

Finally, dummy variables control for systematic differences between districts. ZT-related CSISA activities started in Bhojpur district, which serves as the base district in the models. Since all statistically significant district effects are negative and quite substantial in magnitude (11 to 25 percentage points), they are likely related to the shorter time of exposure to ZT technology and the lag in the development of the respective service economy.

## Discussion

Based on the census data collected in our 40 study villages, we find that the adoption rate of ZT wheat was 27.5% in the 2012/13 *rabi* season. However, since these villages were randomly selected from a population of villages with CSISA’s direct intervention(cf. section “Research area, sampling procedure, and data collection”), the ZTadoption rate outside this population is likely to be lower. This hypothesis is supported by the great variation in adoption rates among the studied villages, ranging from 1% to 93%, which indicates that the diffusion of ZT is still in an early stage when the intensity of project effortsmay significantly affect local uptake. Nevertheless,we find that the structural attributes of the 40 survey villages did not differ significantly from a random sample of 140 villages in the same districts in terms of access to all-weather roads, agricultural input and output markets, and agricultural extension centers. This suggests that, apart from the project intervention, the survey villages can be considered representative of all villages in the six target districts.

At the time of the survey, only 44.3% of sample farmers were aware of ZT to an extent that would enable an informed decision about whether or not to adopt the technology. Again, this illustrates that ZT technology is still in a relatively nascent stage of diffusion in the area and highlights that the adoption rate of 27.5% within the research villagesmust be viewed as a snapshot in time rather than a final assessment. Furthermore, the relatively low awareness rate justifies our modeling approach that accounts for the fact that non-exposed households had no chance to adopt the technology, although they may have done so if they had known about it. Among the sub-sample who knew about ZT, two out of three farmers chose to use it. However, we find a distinct scale bias both with respect to knowledge of ZT and the actual use of the technology, which is evident from our descriptive results (Table 2), as well as from our econometric analysis; the latter shows that there is a quadratic positive relationship between farm size and ZT awareness and adoption, with the magnitude of the estimated coefficients indicating that the marginal effect would turn negative only beyond a farm size of 8.8 ha^10^ in the awareness stage and 6.9 ha in the adoption stage. Given an average farm size of 1.3 ha (median 0.8 ha) and a 99% percentile of 6.5 ha, this means that the marginal effect of farm size remains positive across the entire range of landholdings usually encountered in the research area. Hence, our econometric analysis confirms the existence of a clear bias in favor of larger farms, but it shows that the bias decreases at the margin, i.e. it is most pronounced among the smallest landholders.

Nevertheless, our data also suggest that the scale bias may diminish over time. As Table 3 shows, the number of ZTadopters grew faster among the smaller than among the larger scale farmers, and once farmers of smaller farms tested the technology, they expanded its use and became full adoptersmore rapidly than those with larger farmers. This suggests that while smaller scale farmers may lag behind larger scale farmers in adopting the new practice, as is often observed with agricultural innovations (Rogers 2003: 267 ff.), they may ultimately catch up. Since our recall-based survey captured a period of three years only, observations on the temporal aspect of ZT adoption should be interpreted with caution. To explore questions of adoption dynamics more reliably, longer-term panel data will be required (cf. Doss 2006). It also has to be kept in mind that for most smallholder farmers in Bihar, access to custom-hire services is a prerequisite to the adoption of ZT technology. Even if small scale farmers were as willing to adopt the technology as larger scale farmers, ZT service providers may give preference to larger customers as the provision of ZT services on small farms is associated with higher per-hectare transaction costs; conversely, with a growing number of ZTservice providers, increasing competition may make them less selective in choosing their clients, hence mitigating the scale bias (Keil et al. 2016). Small plot sizes pose a technical barrier to the use of this tractor-based technology that might ultimately be addressed through expansion of markets for scale-appropriatemachinery; however, our analysis indicates that this was not a binding constraint among the knowledge-exposed sub-population in the 2012/13 *rabi* season. Again, as the use of the technology matures in Bihar, panel data are required to shed light on the growth dynamics of the ZT related service economy and possible changes in the social inclusiveness of such services under more competitive conditions.

The risk involved in an agricultural innovation as well as variations in farmers’ risk preferences have long been shown to affect technology adoption (Feder et al. 1985). Due to the proven short- and long-term benefits of ZT in wheat (Mehla et al. 2000; Erenstein and Laxmi 2008; Chauhan et al. 2012; Gathala et al. 2013; Keil et al. 2015), this is objectively a risk-reducing technology, especially when considering its potential to facilitate earlier crop establishment to avoid terminal heat stress (see below). However, our analysis shows that farmers who are less risk-averse are more likely to be aware of and use ZT. This finding can be explained by considering that ZT technology is an unfamiliar technique contrary to farmers’ traditional practices, which is likely *perceived* to be risky at an initial stage of diffusion (Feder et al. 1985; Rogers 2003: 20 f.). As shown in Table 3, it is interesting to note that the very early adopters of ZT in Bihar used the new technology on 95% of their wheat area; among the later adopters, especially in the largest tercile, our data show a tendency to be slightly more cautious resulting in a smaller scale of adoption. The opposite could be expected, as later adopters should gain confidence from witnessing the performance of the technology in earlier adopters’ fields; our observation could be related to ‘innovators’ in agriculture being ‘venturesome’ in general, whereas subsequent adopters tend to be more risk-averse (Rogers 2003: 282 ff.).

Regarding the role of different communication channels to inform farmers about ZT, we find that access to mass media (radio broadcasts), agricultural extension, and individual social networks are important. As suggested by Rogers (2003: 18) and empirically observed in rural India (Matuschke and Qaim 2009; Songsermsawas et al. 2016), our findings confirm that social networks are generally formed along homophilous lines (Table 4). However, we also find evidence of a certain degree of heterophily, as manifested by significantly larger NM farm sizes as compared to respondents’ own farm sizes within each farm size tercile (Table 2). This ‘upward orientation’ in the choice of NMs is often observed when it comes to communication about agricultural innovations, as there is no information to share if the technical grasp of a new technology is identical among the communicating individuals (Rogers 2003: 19). We find both endogenous and exogenous individual network effects, i.e., it mattered whether NMs had adopted ZT or not, but also the intensity of contact and the educational status of their NMs had an effect on the respondents’ knowledge of ZT and adoption decision. While Matuschke and Qaim (2009) detected endogenous individual network effects with respect to the adoption of hybrid technology in wheat and pearl millet, they found no significant exogenous network effects; our deviating findings may be due to the nature of ZT technology which, from the user’s point of view, is likely perceived as a more dramatic transition from traditional practices, and one that requires more specialized knowledge. In addition to the effects of *individual* social networks, technology adoption is significantly influenced by what other farmers in the village do, as indicated by the village-level adoption rate. Our findings indicate that especially the smaller-scale farmers rely more on their individual networks when they gather information about a relatively complex and, hence, knowledge-intensive technology such as ZT, permitting effective communication with peers of similar socioeconomic status (Rogers, 2003: 205).While the adoption decision of these farmers is also strongly influenced by their individual networks, the larger-scale farmers are encouraged by adoption by their general peerswithin the village rather than their individual NMs.

The finding that delayed monsoon rains spur ZTadoption in wheat is plausible as delayed rains lead to delayed planting and harvesting of rice, thus directly affecting the sowing time of the subsequent wheat crop. Since, in the eastern IGP, late establishment of wheat can entail yield penalties up to 50% due to terminal heat stress (Mehla et al. 2000; Erenstein and Laxmi 2008; Chauhan et al. 2012; Gathala et al. 2013), farmers have a strong incentive to establish their wheat crop as quickly as possible. This is facilitated by the use of ZT as compared to conventional tillage which involves multiple passes with the tractor for ploughing and planking (Erenstein and Laxmi 2008). However, making effective use of the time-saving potential of ZT requires the existence of a relatively dense network of ZT service providers. To date, ZT services are not always available on time (cf. section “Farmers’ stated motivating factors for zero-tillage adoption and evaluations of the practice”), and our regression analysis shows that the availability of a service provider in close proximity is an important prerequisite to the use of the technology.

Comparing the simple probit models 1 and 2 with their respective heckprobit counterparts 3 and 4, we find that the latter lead to only minor improvements in the explanatory power in terms of correctly predicted outcomes. For some key determinants of ZT adoption, such as farm size, education, risk preferences, and endogenous individual network effects among the smallest farm size tercile, non-exposure bias is quite limited, resulting in rather similar estimates produced by Models 1 and 2 and by the adoption stages of Models 3 and 4, respectively. The reason for this is that these factors have similar effects on both knowledge-exposure and adoption conditional on exposure. However, the proximity of ZT service providers is another key factor which is found to not affect knowledge acquisition, but has a decisive effect on the use of ZTamong the knowledgeable subgroup; in this case, the estimates produced by the simple probit models are downward biased, leading to a potential underestimation of the relevance of this factor. Furthermore, the heckprobit specifications offer additional insights into the *process* of adoption, highlighting, for instance, the effects of radio ownership, relative scarcity of family labor, and credit access on farmers’ exposure to agricultural innovations. The comparison of Models 1 and 3 with Models 2 and 4 shows that the inclusion of individual social network characteristics leads to some improvement in model sensitivity, i.e. it increases the share of correctly predicted ZTadopters by 7.1 percentage points. More importantly, however, their inclusion sheds light on the differential effects of farmers’ individual social networks and their general peers on gaining knowledge about an innovation and deciding about its use, from which practical recommendations for a more effective ZT related extension approach can be derived.

## Conclusions and recommendations

In this study we investigated determinants of farmers’ adoption of ZT wheat in Bihar. Previous research found the use of ZT to be a promising technology for sustainable wheat intensification in the eastern IGP where yields are particularly low. Apart from longer-term benefits, immediate cost-savings as compared to conventional tillage are expected to make the technology attractive to farmers. However, adoption of ZT is complicated by two factors, namely (i) that many farmers do not know about the technology and (ii) that the large majority of farmers rely on custom-hire services to access the technology. This study contributes to the existing body of literature in two ways. First, it is the first such assessment in the eastern IGP where the diffusion of ZT is still in a relatively nascent stage; since highly significant productivity enhancing and cost-reducing effects of ZT have been confirmed in this densely populated and poverty-stricken area, an assessment of adoption determinants is urgently needed for deriving practical recommendations to foster its uptake in view of the pressing need to close yield gaps for enhanced food security. Second, this article makes a methodological contribution by examining the role of different information channels – including farmers’ individual social networks – in two stages of the adoption process: learning about the new technology (the awareness stage) and deciding whether or not to adopt once knowledge has been gained (the adoption stage).

Although the study was confined to a random sample of villages with at least some ZTadoption, only 44.3%of sample farmers knew about the technology. This clearly justified our modeling approach that accounted for potential non-exposure bias, which was manifested in an underestimation of the importance of proximate service providers for ZTadoption in the simple probit models. Furthermore, differentiating the two stages of knowing about ZT and adopting the technology, and accounting for social network effects in both stages, led to improved explanatory power and important insights with respect to the *process* of adoption, with practical implications for ZT related extension efforts.

We documented a substantial scale bias in favor of largerscale farmers regarding ZT related knowledge acquisition. While our analysis indicates that the mass media (radio broadcasts) can be an effective means to raise farmers’ awareness of an agricultural innovation, farmers’ individual social networks, which are mostly confined to their own village, play an important role for knowledge acquisition as well. This is especially true for the smaller-scale farmers who have limited access to agricultural extension and other formal sources of agriculture related messaging. However, our analysis confirms previous research findings that social networks are formed along homophilous lines, i.e. there is limited social interaction across socioeconomic strata within a village. This means that agricultural development strategies that attempt to diffuse extension messages through ‘progressive’ farmers, who usually belong to the better-off, better-educated, and higher-caste stratum, have limited scope of reaching the poorer segments in a village.

Farmers’ relatively low level of awareness of ZT illustrates that, at the time of the survey, the technology was still in a relatively nascent stage of diffusion in the area. Therefore, the adoption rate of 27.5% within the study villages should be viewed as a snapshot in time rather than a final assessment. Among the sub-sample who knew about ZT, two out of three farmers chose to use it. However, similar to the awareness stage, we also found a distinct scale bias with respect to the use of ZT, conditional on being aware of the technology. Hence, in tendency, larger scale and better educated farmers are more likely to know about and adopt ZT. Based on recall data spanning a three-year period, we found some indication that this scale bias may diminish over time, but panel data will be required to investigate the development of the social inclusiveness of the technology more reliably.

We conclude that:

First, there is need for further awareness-raising campaigns regarding ZT technology; this should include the use of mass media (especially radio messages), but be supplemented by extension messages targeted at contact farmers representing different social strata to allow effective within-village diffusion via social networks. The latter component is likely to be of particular importance for the diffusion of knowledge-intensive technologies, such as ZT, among the poorer strata.

Second, in order to reduce the observed scale bias favoring larger scale farmers, the network of ZT service providers needs to be further expanded, and the transaction costs of servicing smaller scale farmers need to be reduced through more complex business models involving demand aggregation and service coordination.

Third, since, in the eastern IGP, late wheat sowing can entail yield penalties up to 50% due to terminal heat stress, the time-saving potential of ZT in wheat establishment appears to be valued by farmers, especially under conditions of increasingly unreliable monsoon rains resulting in delayed harvest of the preceding rice crop. This time-saving aspect of ZT should be further highlighted in awareness raising and training activities as a means to reduce the risk of heat-stress-related yield losses in wheat.

## Footnotes

1 Since differences in time of planting and the use of fertilizer, herbicides, and other inputs between ZTand conventional-tillage wheat were controlled for in the analysis, the yield gain was attributed mainly to reduced evaporative losses of soil water under deficit irrigation conditions; see Keil et al. (2015) for details.

2 Based on village survey data collected in 2008.

3 Household heads weremale in 98%of cases. Due to the non-sensitive nature of the research topic, the male enumerators did not face any problem in interviewing the few female household heads. The enumerators worked for a New Delhi-based consulting firm specialized in research and communications in the social and agricultural sectors.

4 The maximum likelihood procedure does not directly estimate *ρ* but *atanh* $\rho =\frac{1}{2}\ln(\frac{1+\rho }{1-\rho }).$

5 ‘Primary’ here is defined as being cited by at least 10% of respondents.

6 On a scale from 1 (= very small problem) to 5 (= very severe problem), the average ratings ranged from 4.1 to 4.3 among the largest landholding tercile and from 3.2 to 3.6 among the smallest tercile (Mann-Whitney tests significant at *P* < 0.01).

7 Irrigation is a prerequisite to growing wheat during *rabi* season in Bihar.

8 While ZT involves one single pass with the tractor, multiple passes are required for ploughing and planking in the case of conventional tillage (Erenstein and Laxmi 2008).

9 A quadratic relationship was also tested, which produced the expected positive sign on the marginal effect of age and a negative sign on its squared term, but lacked statistical significance in any of the model specifications.

10 Condition for maximum fulfilled for Cultivable area = 0.1859342/0.0211698 = 8.78 ha (rounded coefficients are shown in Table 5).
